# Etching Chemistry Process Optimization of Ethylene Diluted with Helium (C_2_H_4_/He) in Interconnect Integration

**DOI:** 10.3390/mi15121439

**Published:** 2024-11-28

**Authors:** Hwa-Rim Lee, Eun-Su Jung, Jin-Uk Yoo, Tae-Min Choi, Sung-Gyu Pyo

**Affiliations:** School of Integrative Engineering, Chung-Ang University, 84, Heukseok-ro, Dongjak-gu, Seoul 06974, Republic of Korea; ghkfla0725@naver.com (H.-R.L.); eunsuj@cau.ac.kr (E.-S.J.); wlsdnr5771@naver.com (J.-U.Y.); c79411@gmail.com (T.-M.C.)

**Keywords:** metal etch, polymer, Decoupled Plasma Source (DPS), ion chromatography (ICG), Transmission Electron Microscopy (TEM), Auger Electron Spectroscopy (AES)

## Abstract

This study explores the effects of different passivation gases on the properties of polymers formed on aluminum (Al) sidewalls during the etching process in Al-based interconnect structures. The research compares the use of nitrogen (N_2_) and ethylene diluted with helium (C_2_H_4_/He) as passivation gases, focusing on the resulting polymer’s composition, thickness, and strength, as well as the levels of residual chlorine post-etch. The findings reveal that using C_2_H_4_ leads to the formation of a thinner, weaker polymer with lower chlorine residue compared to the thicker, stronger polymer formed with N_2_. Elemental analysis further highlights significant differences in carbon and oxygen content, with C_2_H_4_-based polymers exhibiting lower carbon and higher oxygen levels. These results underscore the critical impact of passivation gas choice on the etching process and the integrity of Al-based interconnects, offering valuable insights for optimizing metal etching processes in semiconductor manufacturing.

## 1. Introduction

Ion-assisted anisotropic etching, currently utilized in the metal etch process, operates via the surface inhibitor mechanism [1,2]. This mechanism provides sufficient energy for low-energy ions to remove the nonvolatile polymer layer (surface inhibiting or blocking layer) deposited on the etched surface. In regions where the polymer deposition occurs without ion bombardment, the blocking layer is not removed, thereby protecting these areas from reactive gas etching. Energetic particles incident on the wafer are typically oriented perpendicularly to the wafer surface, striking the bottom of the etched regions. However, the sidewalls of the etched features receive little to no impact from these energetic particles. The gas responsible for creating the nonvolatile polymer layer is referred to as the passivation gas [3,4,5,6,7].

In the aluminum (Al) etch process, gases containing chlorine or chlorine itself are primarily used as reactive gases [8,9,10,11]. Aluminum (Al) corrosion occurs post-etch due to the hydrolysis of chlorine or chlorine-containing residues (predominantly AlCl_3_) left on the Al sidewall, substrate, or photoresist. These residues absorb moisture, forming HCl, which corrodes Al, creating additional AlCl_3_. This corrosion reaction continues as long as moisture is present [12]. In the Al etch process, N_2_ gas has traditionally been used as the passivation gas. While N_2_ enables the achievement of a stable profile and ensures process stability, it also presents challenges such as increased chamber particles, polymer residue, and corrosion due to the formation of a hard polymer [13]. In Kiryukhantsev-Korneev et al., using the pulsed cathodic arc evaporation (PCAE) method with a TiC-NiCr-Eu_2_O_3_ cathode, protective coatings were deposited in Ar, N_2_, and C_2_H_4_ environments, and their properties were evaluated. The coating generated in the N_2_ environment exhibited the lowest corrosion current density, at 0.012 µA/cm^2^. Additionally, the coating produced in the C_2_H_4_ environment demonstrated a low friction coefficient (0.35) and excellent wear resistance. Through a literature review on passivation gases, it was found that the new gas, C_2_H_4_, offers superior process characteristics compared to N_2_ [14,15]. Therefore, in this study, C_2_H_4_ was applied to the CMOS image sensor etch process.

This study aims to systematically investigate the effects of various passivation gases, specifically nitrogen (N_2_) and ethylene diluted with helium (C_2_H_4_/He), on the properties of polymers formed on Al sidewalls during the etching process. This includes an analysis of the differences in polymer strength, thickness, and chemical composition, as well as the levels of residual chlorine that remain post-etch. This study aims to provide insights into how these factors influence the overall etch process performance and the integrity of Al-based interconnect structures, ultimately contributing to the optimization of metal etching processes in semiconductor manufacturing. To compare the corrosion stability, the residual chlorine levels when using N_2_ and C_2_H_4_ as passivation gases were evaluated. This was achieved by rinsing the wafer with deionized (DI) water and then determining the ion composition of the water using ion chromatography (ICG) [16,17]. Since chlorine dissolves in water as an anion, its quantity on the wafer can be determined through anion analysis [18]. For precise analysis via ICG, wafers must be separated and sealed individually after each process step.

## 2. Materials and Methods

This study aimed to evaluate the effects of different passivation gases on the metal etching process, specifically focusing on the sidewall polymer formation and residual chlorine levels in Al-based interconnection structures. The experiments were conducted using two passivation gases: N_2_ and C_2_H_4_/He.

### 2.1. Fundamental Characteristic Evaluation

The fundamental characteristic evaluation when using N_2_ and C_2_H_4_/He as passivation gases is shown in Table 1. The specific experimental conditions used for the etching for fundamental characteristic evaluation are as follows.

The chamber pressure during etching was maintained at 8 mTorr. The source power was set to 1200 W, and the bias power to 160 W. Chlorine (Cl_2_) gas was supplied at a flow rate of 80 sccm, while boron trichloride (BCl_3_) was introduced at 30 sccm. Depending on the passivation gas under evaluation, either nitrogen N_2_ or C_2_H_4_/He was supplied at a flow rate of 15 sccm. The etching duration was fixed at 30 s. These conditions were specifically optimized to analyze the impacts of the different passivation gases on polymer characteristics and etching residues.

Notably, the mean wafers between cleans (MWBC) when using C_2_H_4_/He was approximately seven times higher than that when using N_2_. This is because the polymer component that adheres to the chamber wall or dome when using C_2_H_4_/He exhibits different properties than when using N_2_, allowing it to be effectively removed by in situ chamber cleaning (ICC).

### 2.2. Etching Process

In this paper, in addition to the previously evaluated data, the polymer properties and composition of wafers subjected to a passivation gas-based metal etch using N_2_ or C_2_H_4_, followed by a PR strip and a second metal etch, were analyzed and compared. The etching was performed on a stack structure consisting of Si/TEOS/Ti/Al/Ti/TiN, and the photoresist mask utilized was an SR540 (Sartomer Americas, Exton, PA, USA) with a thickness of 9300 Å. The metal thickness of Si/TEOS/Ti100/Al3500/Ti50/TiN600 is 4300 A target, and the photoresist thickness is 9300 A, so it has a selectivity of about 2 to 1. The etching process was conducted in a decoupled plasma source (DPS) etcher (Jusung, Gwangju, Republic of Korea) under the conditions specified in Table 2, with different parameters applied for N_2_ and C_2_H_4_/He gases.

Post-etch stripping was performed using a combination of CF_4_ and O_2_ gases to remove the remaining photoresist and any polymers formed during the etch. Post-etch stripping was performed using a two-step process to remove the remaining photoresist and any polymers formed during the etch. Initially, CF_4_ at 750 sccm and H_2_O at 750 sccm were applied at 1.5 Torr with 1000 W power for 40 s at 260 °C. Subsequently, O_2_ gas at 4500 sccm was introduced at the same pressure with 1400 W power for 80 s. The final step involved H_2_O at 750 sccm under identical conditions but for 30 s. This comprehensive stripping process ensured minimal residue on the wafer.

BCl_3_ was determined based on the PR selectivity and etch residue analysis results. BCl_3_ generally affects the Al etch rate non-uniformity and Al PR selectivity, increases sidewall smoothness, increases line profile slope, and increases CD bias. It is the optimal condition for equipment and process time control by increasing the residue size and density and increasing the overetch time. The stack structure of the wafer and the etching conditions of the plasma chamber in terms of pressure in mT, source (W_s_) and bias power (W_b_) in watt, and gas composition in terms of gas flows (in sccm) and etching time (in s) are reported in Table 2 as Metal Etch.

### 2.3. Analysis Method

CD-SEM (ASML, Veldhoven, The Netherlands) was used to analyze the sidewall profile after the etching and stripping processes. ICG (AD25, Dionex, Sunnyvale, CA, USA) was employed to quantify the residual chlorine on the wafer surface after stripping. To analyze the composition of the polymer deposited on the metal sidewalls, transmission electron microscopy (TEM) (FEG-TEM 200kV, JEOL, Tokyo, Japan) combined with electron energy loss spectroscopy (EELS) (JEOL, Tokyo, Japan) was employed. EELS utilizes the inelastic scattering of incident electrons that lose energy when colliding with inner-shell electrons of the sample, with peaks forming when this energy loss exceeds a critical threshold. The results are displayed as element-specific maps [19,20]. Another analytical method is Auger electron spectroscopy (AES) (PHI680 System, Philips, Amsterdam, The Netherlands). When an X-ray or electron beam irradiates the sample, an electron from an inner orbital is ejected, creating a hole that is filled by an electron from an upper orbital. The binding energy difference is dissipated either as an X-ray or by emitting another electron. For transitions involving electron orbitals with binding energies below 2 keV, the process is predominantly governed by Auger electron emission. The kinetic energy of the Auger electron is unique to each element and is expressed as E_KL1L2_ = E_K_ − E_L1_ − E_L2_ − Φ_S_, where E_KL1L2_ represents the kinetic energy of the Auger electron resulting from the KL1L2 transition, E_K_, E_L1_, and E_L2_ denote the binding energies of the K, L1, and L2 orbitals, respectively, and Φ_S_ represents the work function of the spectrometer [21,22,23].

## 3. Results and Discussion

### 3.1. Polymer Formation and Morphology After Metal Etching and Stripping

**Figure 1:**
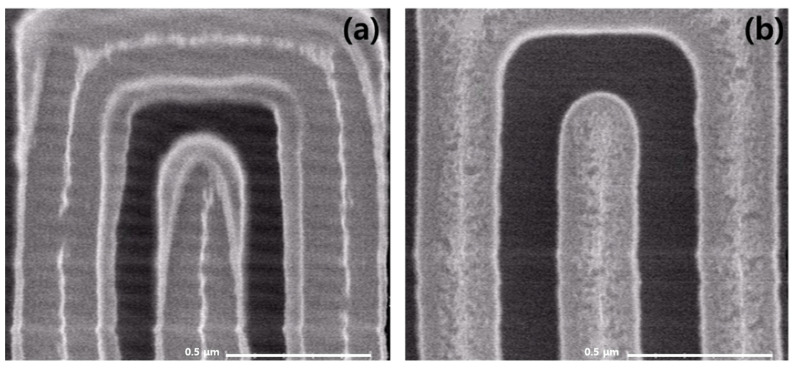
CD-SEM image of a wafer that has been metal etched and stripped using (**a**) N_2_ and (**b**) C_2_H_4_/He as passivation gases.

Figure 1 shows a wafer that has been metal etched and stripped using N_2_ or C_2_H_4_/He as a passivation gas, which was confirmed by critical dimension scanning electron microscopy (CD-SEM). In the case of N_2_, it can be seen that a hard polymer was formed and remained in the form that surrounded the photoresist even after the photoresist was removed through the strip. On the other hand, in the case of C_2_H_4_/He, a weak polymer was formed, and it can be seen that it was thinly laid on the top layer of metal after stripping.

**Figure 2:**
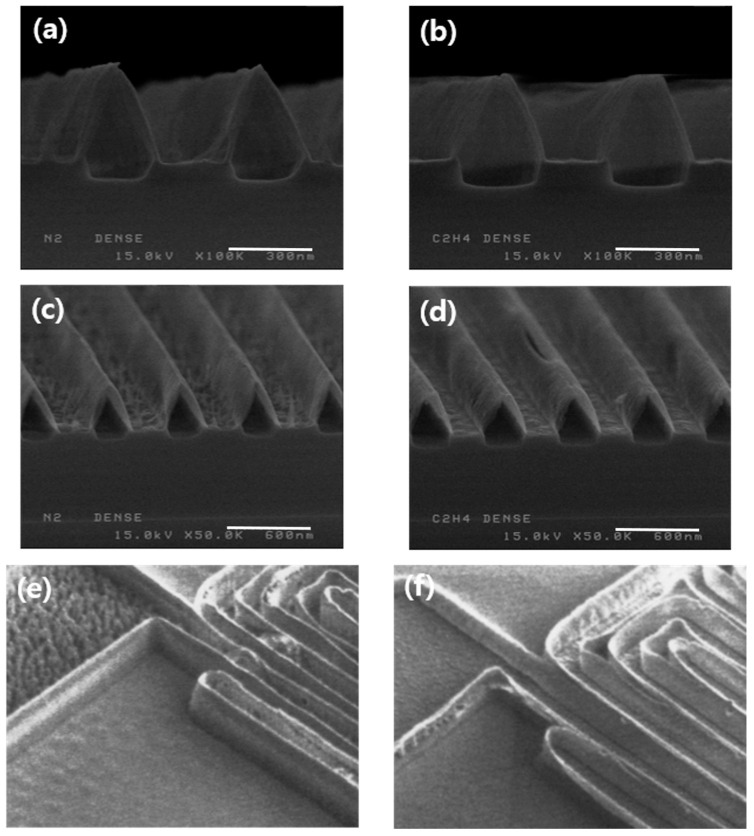
SEM image of a wafer that has been metal etched, stripped, and 2nd metal etched using (**a**,**c**,**e**) N_2_ and (**b**,**d**,**f**) C_2_H_4_/He as passivation gases.

Figure 2 shows a wafer that was metal etched using N_2_ or C_2_H_4_/He as a passivation gas to check the polymer morphology, and the strip and second metal etch were confirmed by SEM. The second metal etch was stopped after the main etch and end point detection (EPD) because over-etching can distort the shape of the remaining polymer [24,25]. Therefore, the place where the metal was before the second metal etch remains bulging because little oxide is lost, and there is also a lot of residue. In the case of N_2_, the residue remains lumpy, while in the case of C_2_H_4_/He, it is thinly spread out.

### 3.2. Residual Chloride and Fluorine Analysis

**Figure 3:**
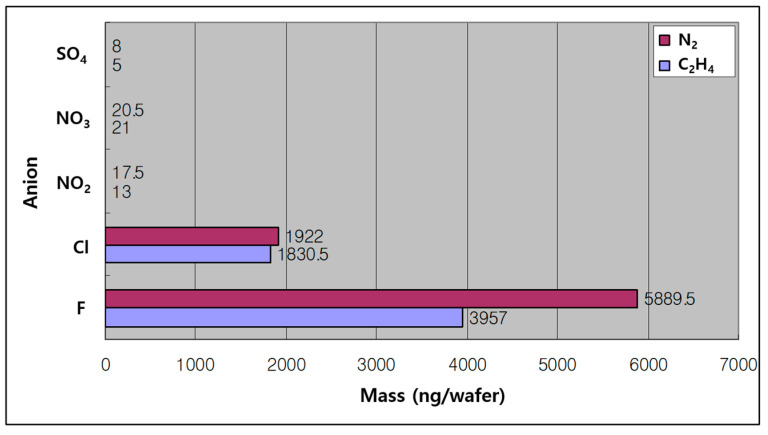
ICG analysis results for wafers that have been metal etched and stripped using N_2_ or C_2_H_4_/He as the passivation gas.

We evaluated the amount of chlorine remaining in the wafers after metal etch and PR strip using N_2_ or C_2_H_4_/He as the passivation gas. The ICG analysis results in Figure 3 show that the amount of F is high when using both N_2_ and C_2_H_4_/He, which is due to the relatively large amount of CF_4_ gas used during the post-etch strip process. However, it can be seen that the residual amount of F is high in the case of the wafer that was etched using N_2_ as a passivation gas after the same strip process. The higher residual amount of F in the case of N_2_ was assessed to be due to the fact that the hard polymer surrounds the metal border like a film, resulting in a larger surface area where F can remain. Chlorine is introduced through the use of chlorine-based gases (e.g., Cl_2_ and BCl_3_) during the etching process. These gases react with the aluminum (Al) in the metal stack, forming chlorinated compounds such as AlCl_3_, which can leave residual Cl on the wafer surface. There is not much difference when comparing the Cl residue, but there is slightly more with N_2_. If the intention was to measure the residual Cl solely after the etching process, the strip procedure should have been avoided. However, since all processes using DPS perform strip after etching, it is appropriate to analyze the wafer after stripping for process evaluation. Although sulfur was not specifically introduced as a primary gas, it could be present as a trace element or impurity within the etching gases or equipment. Another potential source might be residual contamination from previous processes or the materials in the chamber.

### 3.3. Elemental Composition of Polymer Deposits

**Figure 4:**
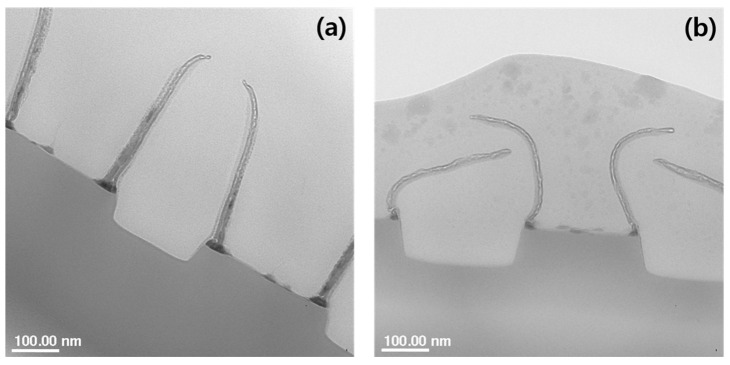
TEM image of a wafer that has been metal etched, stripped, and 2nd metal etched using (**a**) N_2_ and (**b**) C_2_H_4_/He as passivation gases.

Figure 4 is a TEM image of the wafer after the metal etch, PR strip, and second metal etch using N_2_ and C_2_H_4_/He as passivation gases. In the case of using N_2_, the thickness of the polymer is thick and stands upright, while in the case of using C_2_H_4_/He, the thickness of the polymer is thin and collapsed. This shows that less polymer is deposited on the metal side wall and the strength of the polymer is weaker when C_2_H_4_/He is used than when N_2_ is used.

**Figure 5:**
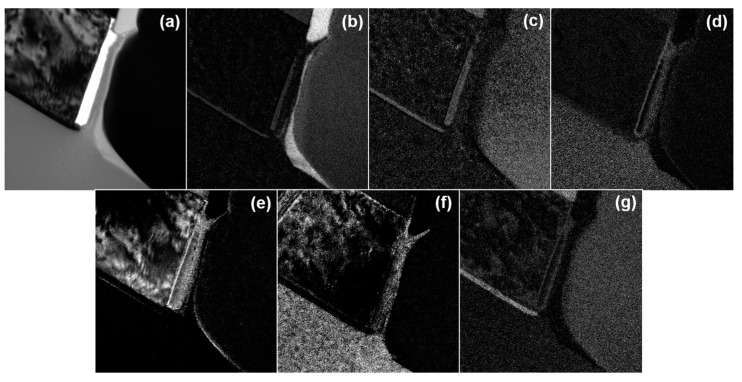
EELS analysis results of a wafer metal etched using N_2_ gas: (**a**) exposure; (**b**) C; (**c**) N; (**d**) O; (**e**) Al; (**f**) Si; and (**g**) Ti.

**Figure 6:**
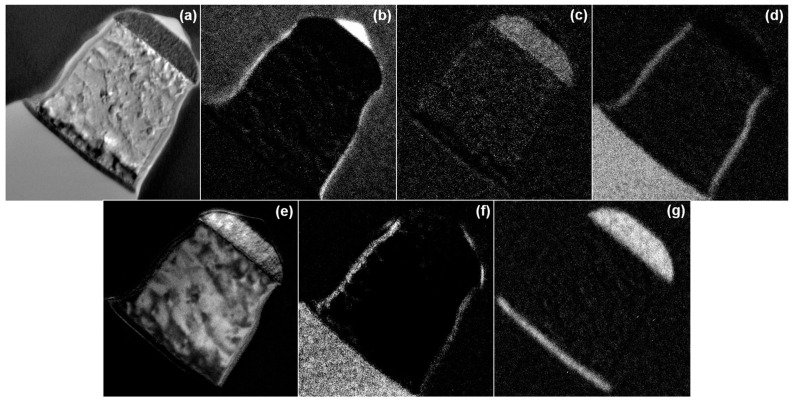
EELS analysis results of a wafer metal etched using C_2_H_4_/He gas: (**a**) exposure; (**b**) C; (**c**) N; (**d**) O; (**e**) Al; (**f**) Si; and (**g**) Ti.

EELS, an adjunct to the TEM, was used to obtain a map of the components. This wafer was only etched with N_2_ or C_2_H_4_/He as a passivation gas and not stripped, so the sample still has photoresist on it. Ion milling and FIB are the two methods of fabricating TEM samples, but since ion milling uses acetone to fabricate the sample, it can dissolve the PR, so we used FIB. For FIB, there is a method with and without a carbon sheet, but we used the method without a carbon sheet because we would see C through EELS analysis. The results of the analysis are shown in Figure 5 and Figure 6. The profile shows a more positive slope in the case of N_2_ (Figure 5a) than in the case of C_2_H_4_/He (Figure 6a). The elements analyzed by EELS were the materials that comprised the wafer and the gases used in the process. The elements identified as being on the Al sidewall were C, O, and Si. In the case of using N_2_ in Figure 5, the map is less reliable for Al and Si than the analysis results under C_2_H_4_/He conditions because Al is not well displayed where Al should be and Si appears. We tried to get a map to confirm whether Al and Si were present in the side walls by EELS analysis, but we could not obtain a definite result.

**Figure 7:**
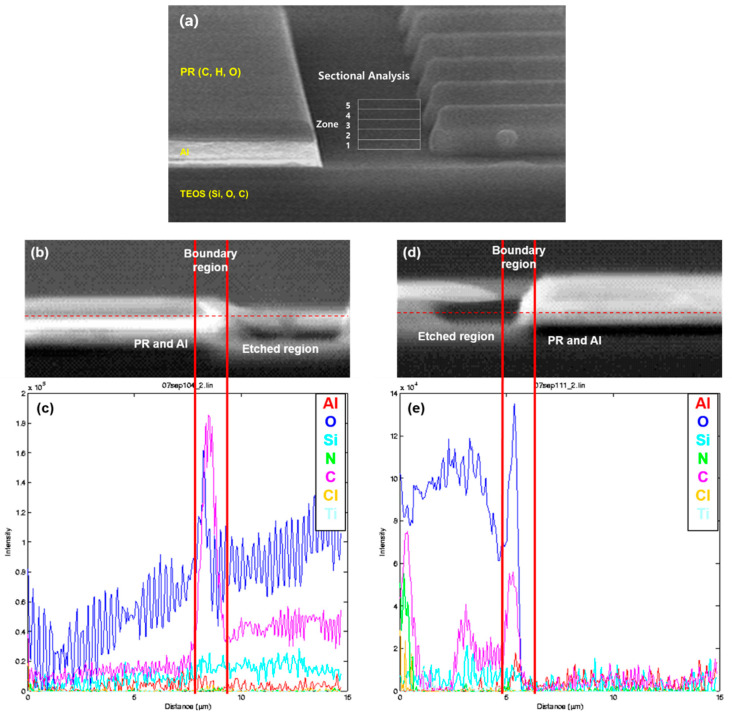
(**a**) AES analysis location; (**b**) image of boundary region etched using N_2_; (**c**) line scan result of Al cross section in (**b**); (**d**) image of boundary region etched using C_2_H_4_/He; and (**e**) line scan result of Al cross section in (**d**).

AES analysis can reveal the elemental composition at the desired location. However, to perform AES analysis, the metal line height needs to be higher than the probing beam spot size of AES. If the metal line height is too low, the analysis results will be inaccurate because unwanted parts of the sample may be included in the analysis results. Therefore, as shown in Figure 7a, the sample with Al height increased to 8000 Å was analyzed. The sample was not stripped, so the photoresist remains. In Figure 7c,d, from the line scan analysis of the Al cross section in both the C_2_H_4_/He and N_2_ cases, peaks of C and O can be found at the boundary of the etched area and the area with PR and Al, i.e., the Al side wall. This shows that C and O were the main components deposited on the Al side wall. The other elements do not show significant changes in their distribution at the boundary. The problem with this result is the distribution graph of Al, which is thought to be caused by the fact that the Al cross section is pointed out, and the Auger electrons are not directed to the detector.

**Figure 8:**
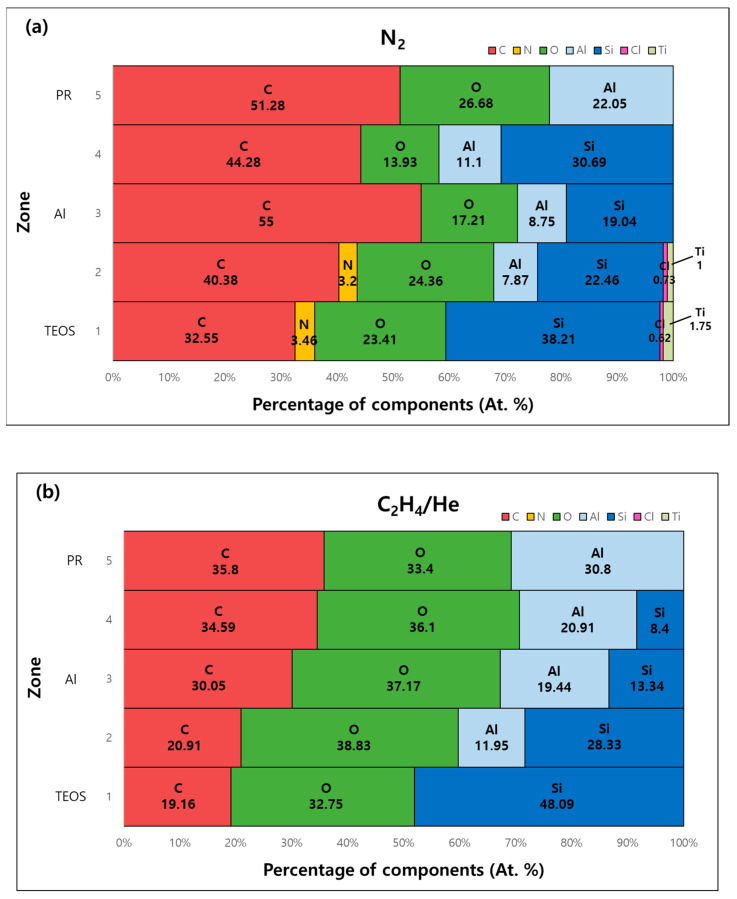
The percentage of components by Al sidewall region using (**a**) N_2_ and (**b**) C_2_H_4_/He as passivation gases.

The AES analysis shows that the components deposited on the Al sidewall were mainly C, O, Si, and Al. As shown in Figure 8, the composition of the material deposited on the Al sidewall was more C and relatively less O, Si, and Al when N_2_ was used. N was also detected at the bottom of the metal. On the other hand, when C_2_H_4_/He was used, O accounted for a more significant proportion, and C, Al, and Si constituted the polymer of the metal sidewall, whereas N was not detected. The common point is that the proportion of Al decreased from the top to the bottom of the metal line.

## 4. Conclusions

The results clearly indicate distinct differences in the polymer formation and residue characteristics when using N_2_ versus C_2_H_4_/He as passivation gases during the metal etching process. N_2_ leads to a more robust, thicker polymer layer that retains more residual fluorine, likely due to its larger surface area and film-like structure along the metal borders. This thick polymer, while protective, may contribute to increased contamination risk due to the higher residue retention.

On the other hand, C_2_H_4_/He results in a thinner, weaker polymer layer, which correlates with lower chlorine and fluorine residues. This suggests that C_2_H_4_/He might be more effective in processes where minimal residue and cleaner sidewalls are required, although the trade-off is a less protective polymer that may offer less mechanical stability. In addition, the resulting polymer layer might be thinner and weaker due to the lower bond strength in the hydrocarbon matrix and a lack of nitrogen-induced cross-linking.

EELS and AES analyses revealed that the elemental composition of the polymer varies significantly between the two gases, with N_2_ leading to a carbon-rich polymer, while C_2_H_4_/He results in a polymer with a higher oxygen content. This compositional difference may influence the etching process’s overall performance, particularly in terms of polymer removal and post-etch cleaning.

In conclusion, the choice of passivation gas plays a critical role in determining the polymer characteristics and residue profiles after metal etching. N_2_ provides a stronger, thicker polymer with higher residue retention, while C_2_H_4_/He offers a cleaner surface with thinner polymer layers. These findings are summarized in Table 3, highlighting the trade-offs between residue retention and polymer strength depending on the passivation gas used.

## Figures and Tables

**Table 1:** Evaluation of the fundamental properties of N_2_ and C_2_H_4_/He as passivation gases.

STACK	Si/TEOS/Ti 100/Al 3500/Ti 50/TiN 600	
MASK	SR540 9300 Å	
ETCH	8 mT/1200 Ws/160 Wb/80 Cl_2_/30 BCl_3_/15 (N_2_/C_2_H_4_/He)/30 s	
Passivation Gas	N_2_	C_2_H_4_/He
Etch Rate (Å/min)	6471	5290
Photo Resist Erosion Rate (Å/min)	3969	3523
Selectivity (Aluminum/Photo Resist)	1.66	1.51
MWBC (Mean Wafers Between Cleans)	~700	~4995

**Table 2:** The stack structure of the wafer and the detailed parameters of the etch process using N_2_ and C_2_H_4_/He gas.

STACK	Si/TEOS/Ti 100/Al 3500/Ti 50/TiN 600	
MASK	SR540 9300 Å	
Metal Etch (DPS) (Pressure/Source Power/Bias Power/Cl_2_ Flow Rate (sccm)/BCl_3_ Flow Rate (sccm)/N_2_ or CH_4_ Flow Rate (sccm)/Duration)	(N_2_) 5E05-3B 8 mT/800 W_s_/140 W_b_/80 Cl_2_/40 BCl_3_/40 N_2_/EPD 7 mT/800 W_s_/180 W_b_/50 Cl_2_/50 BCl_3_/20 N_2_/15 s 8 mT/800 W_s_/120 W_b_/60 Cl_2_/40 BCl_3_/10 N_2_/10 s	(C_2_H_4_/He) 5E05-3A 8 mT/800 W_s_/140 W_b_/80 Cl_2_/40 BCl_3_/20 C_2_H_4_/He/EPD 7 mT/800 W_s_/180 W_b_/50 Cl_2_/50 BCl_3_/15 C_2_H_4_/He/20 s 8 mT/800 W_s_/130 W_b_/60 Cl_2_/40 BCl_3_/15 C_2_H_4_/He/30 s
Strip (Duration/Pressure/H_2_O Flow Rate (sccm)/CF_4_ Flow Rate (sccm)/Power)	40 s/1.5 T/750 H_2_O/750 CF_4_/1000 W (260 °C) 80 S/1.5 T/4500 O_2_/1400 W 30 S/1.5 T/750 H_2_O/1000 W	
2nd Metal Etch (DPS) (Pressure/Source Power/Bias Power/Cl_2_ Flow Rate (sccm)/BCl_3_ Flow Rate (sccm)/N_2_ or CH_4_ Flow Rate (sccm)/Duration)	(N_2_) 8 mT/800 W_s_/140 W_b_/80 Cl_2_/40 BCl_3_/40 N_2_/EPD	(C_2_H_4_/He) 8 mT/800 W_s_/140 W_b_/80 Cl_2_/40 BCl_3_/20 C_2_H_4_/He/EPD

**Table 3:** Comparing the results of metal etching with N_2_ and C_2_H_4_/He as passivation gases.

Passivation Gas	N_2_	C_2_H_4_
Chlorine Residue	1922	1830.5
Polymer Strength	Strong	Weak
Polymer Thickness	Thick	Thin
Carbon Content in Polymer	55.00%	30.05%
Oxygen Content in Polymer	17.21%	37.17%
Silicon Content in Polymer	08.75%	13.34%
Aluminum Content in Polymer	19.04%	19.44%

## Data Availability

The original contributions presented in the study are included in the article; further inquiries can be directed to the corresponding author.
